# Complete Genome Sequence of *Wohlfahrtiimonas chitiniclastica* Strain SH04, Isolated from *Chrysomya megacephala* Collected from Pudong International Airport in China

**DOI:** 10.1128/genomeA.00119-13

**Published:** 2013-04-04

**Authors:** Xiao-Mei Cao, Tuo Chen, Li-Zhi Xu, Li-Si Yao, Jun Qi, Xiao-Long Zhang, Qing-Li Yan, Yao-Hua Deng, Tian-Yu Guo, Jing Wang, Kong-Xin Hu, Bao-Liang Xu

**Affiliations:** a Institute of Health, Chinese Academy of Inspection and Quarantine, Beijing, China; b Beijing Macro & Micro Test Biotech Company, Beijing, China; c Tianjin Entry-Exit Inspection and Quarantine Bureau, Tianjin, China; d Shanghai Entry-Exit Inspection and Quarantine Bureau, Shanghai, China

## Abstract

*Wohlfahrtiimonas chitiniclastica* bacilli that live in the larvae of a parasitic fly were recently isolated and are speculated to be the cause of fulminant sepsis. Here we report and analyze the complete genome sequence of *Wohlfahrtiimonas chitiniclastica* strain SH04. No complete genome sequence of a *Wohlfahrtiimonas chitiniclastica* isolate has been documented previously.

## GENOME ANNOUNCEMENT

*Wohlfahrtiimonas chitiniclastica* is phylogenetically close to *Ignatzschineria larvae* (1), which causes severe wound myiasis in cattle. *I. larvae* was isolated from a homogenate sample of first- and second-stage larvae of the obligate parasitic fly *Wohlfahrtia magnifica* (2). *W. chitiniclastica* was originally isolated from the homogenate of third-stage larvae of the same fly (3). However two cases of myiasis due to *W. chitiniclastica* have been described recently in homeless adults (4, 5). One case was fulminant sepsis that occurred in South America (4). A 70-year-old homeless male patient with a history of alcohol abuse, severe smoking, and occlusive peripheral arteriopathy of the lower limbs covered by multiple erythematous plaques in both inguinal regions died of fulminant sepsis 5 days after admission to the hospital. The culture of the patient’s blood was identified by 16S rRNA sequence as bacilli of *Wohlfahrtiimonas chitiniclastica*. Another case (5) occurred in southeastern France in a 60-year-old homeless woman with a history of alcoholism. She was covered with thousands of body and hair lice, and dozens of insect larvae. Louse-borne infection had been ruled out by data from several experiments. The cause of infection was believed to be *W. chitiniclastica*. Although in these cases no larvae or worms were found in the patient’s groin injury, it could be speculated that the skin lesions where the larvae developed were colonized or infected with the organism, which then entered the bloodstream.

Thus, the bacteria that live in the parasitic fly may be the causative agent of severe infections in homeless patients. We have isolated a strain of *W. chitiniclastica* SH04 from the adult fly of *Chrysomya megacephala* collected from the Pudong International Airport of China in 2011. Here we report and analyze the complete genome sequence of *W. chitiniclastica* strain SH04. No complete genome sequence of a *W. chitiniclastica* isolate has been documented previously.

The complete genome sequence of *W. chitiniclastica* SH04 comprises a total of 6.84 M pair-end reads (500 bp insert size library), totaling 1.23 Gb and more than 500× coverage by Illumina GAII. The genome was assembled by Velvet (6), and the genomic sequence contains a total of 2.12 Mb from 21 large scaffolds (28 contigs), with an average G+C content of 43.48%. It contains 2,006 open reading frames (ORFs) predicted by GeneMarkS (7), 3 rRNAs predicted by RNAmmer-1.2 (8), and 47 tRNA genes predicted by tRNAscan-SE-1.3.1 (9). A total of 1,371 ORF annotations were determined by using BLAST 2.2.26 to search the databases COG, Swiss-Prot, Uniprot, and NR.

16S rRNA gene sequencing of *W. chitiniclastica* SH04 (GenBank accession no. JQ796717) showed 98.66% identity with the sequence corresponding to the *W. chitiniclastica* strain S5 (GenBank accession no. NR042554), which was isolated from third-stage larvae of *Wohlfahrtia magnifica* (Diptera: Sarcophagidae). However, the sequence similarity to the representatives of the *Ignatzschineria* *larvae* strain L1/68 was lower, at 93.57%. The availability of the genome sequence for *W. chitiniclastica* SH04 will be helpful for recognizing and analyzing this strain.

### Nucleotide sequence accession numbers.

The *Wohlfahrtiimonas chitiniclastica* SH04 Whole-Genome Shotgun project has been deposited at DDBJ/EMBL/GenBank under the accession number AOBV00000000. The version described in this paper is the first version, AOBV01000000.
